# Short- and long-term outcomes of laparoscopy-assisted versus open total gastrectomy for gastric cancer: a propensity score-matched analysis

**DOI:** 10.18632/oncotarget.16852

**Published:** 2017-04-05

**Authors:** Jian-Xian Lin, Ju-Li Lin, Chao-Hui Zheng, Ping Li, Jian-Wei Xie, Jia-Bin Wang, Jun Lu, Qi-Yue Chen, Long-Long Cao, Mi Lin, Ru-Hong Tu, Ze-Ning Huang, Chang-Ming Huang

**Affiliations:** ^1^ Department of Gastric Surgery, Fujian Medical University Union Hospital, Fuzhou 350001, Fujian Province, China

**Keywords:** stomach neoplasms, laparoscopy-assisted total gastrectomy, propensity score matching, prognosis

## Abstract

**Background:**

Few studies have been designed to evaluate the short- and long-term outcomes of laparoscopy-assisted total gastrectomy (LATG), and a retrospective study of a large patient cohort is valuable before conducting randomized controlled clinical trials.

**Results:**

Among all patients, age, tumor location, histologic type, pT stage, pN stage and pTNM stage significantly differed between the LATG group and OTG group. After the propensity score matching, the clinicopathological characteristics did not significantly differ between groups. The operation time, estimated blood loss, time to first flatus and the number of retrieved lymph nodes (*P* < 0.05) were better in the LATG group than the OTG group. Morbidity and mortality were lower in the LATG group than the OTG group (*P* < 0.05) for pre-matched patients. However, significant intergroup differences in morbidity were not identified after propensity matching. Although overall survival did not significantly differ between groups for the pre-matched patients, the 3-year cumulative survival rates were significantly lower in the LATG group (89.9%) than the OTG group (97.7%) for patients with stage I disease (*P* = 0.028). After propensity score matching, the analysis of the cumulative survival curve did not show a significant difference for any cancer stage.

**Materials and Methods:**

We prospectively collected data from 1096 patients who underwent total gastrectomy for gastric cancer. Propensity score matching was applied to compare the covariates between the LATG group and the open total gastrectomy (OTG) group. Operative outcomes and long-term outcomes were compared between the two groups.

**Conclusions:**

Implementation of LATG for gastric cancer is a safe, reliable and minimally invasive procedure with long-term outcomes similar to those of OTG. Further randomized controlled clinical trials can be conducted to provide valuable evidence of the safety and efficacy of LATG in treating gastric cancer.

## INTRODUCTION

Since laparoscopy-assisted distal gastrectomy (LADG) for early gastric cancer (EGC) was first reported by Kitano et al. [1] in 1994, the use of laparoscopy-assisted gastrectomy (LAG) has gained popularity as a treatment for EGC. The advantages of minimally invasive laparoscopic gastrectomy include faster recovery, decreased blood loss, fewer postoperative complications and a shorter hospital stay [2–4]. Laparoscopic gastrectomy has been a standard approach for EGC due to its minimal invasiveness and similar long-term outcomes compared with conventional open surgery. Many experienced institutions have already extended the indications of laparoscopic gastrectomy to highly selective patients with advanced gastric cancer (AGC), and the majority of retrospective studies have focused on patients with AGC who underwent laparoscopic distal gastrectomy. Moreover, centers in Japan, Korea and China have been conducting several randomized controlled trials to evaluate the feasibility and safety of LADG for gastric cancer in recent years [5–7]. To the best of our knowledge, few reports have compared the feasibility, safety and long-term outcomes of laparoscopy-assisted total gastrectomy (LATG) to those of open total gastrectomy (OTG), and randomized controlled clinical trials comparing these two modalities have not yet been reported. Therefore, we enrolled a large number of patients and used the propensity score-matching method to reduce bias in our study. We compared the short-term and long-term outcomes achieved by LATG and OTG to investigate the efficacy of the laparoscopic approach for patients with gastric cancer, and the results can provide new evidence to support the application of LATG prior to conducting randomized controlled clinical trials.

## RESULTS

### Patient characteristics

The clinicopathological characteristics of the LATG and OTG groups are shown in Table 1. Among all patients, age, tumor location, histologic type, pT stage, pN stage and pTNM stage significantly differed between the LATG group and OTG group (*P* < 0.05).

**Table 1:** Comparison of clinicopathological features of two groups

Characteristics	All patients (*n* = 1096)		*P*	Patient propensity matching (*n* = 692)		*P*
	LATG (*n* = 510)	OTG (*n* = 586)		LATG (*n* = 346)	OTG (*n* = 346)	
Age	62.3 ± 11.2	60.4 ± 10.2	0.003	61.1 ± 10.0	61.3 ± 10.1	0.952
Gender			0.808			1.000
Male	406 (79.6)	463 (79.0)		274 (79.2)	274 (79.2)	
Female	104 (20.4)	123 (21.0)		72 (20.8)	72 (20.8)	
BMI (kg/m^2^)	21.9±2.8	22.0 ± 2.1	0.395	22.0 ± 2.9	22.0 ± 2.3	0.645
Tumor location			0.001			0.885
Upper	258 (50.6)	341 (58.2)		191 (55.2)	188 (54.3)	
Middle	219 (42.9)	188 (32.1)		136 (39.3)	136 (39.3)	
Total	33 (6.5)	57 (9.7)		19 (5.5)	22 (6.4)	
Tumor size (cm)	5.6 ± 2.7	5.9 ± 2.9	0.083	6.1 ± 3.11	6.0 ± 2.6	0.774
Histologic type			< 0.001			0.920
Differentiated	83 (16.3)	180 (30.7)		60 (17.3)	59 (17.1)	
Undifferentiated	427 (87.7)	406 (69.3)		286 (82.7)	287 (82.9)	
pT stage			< 0.001			1.000
T1	70 (13.7)	54 (9.2)		34 (20.2)	34 (21.1)	
T2	49 (9.6)	49 (8.4)		25 (20.2)	25 (21.1)	
T3	148 (29.0)	78 (13.3)		63 (16.9)	63 (13.6)	
T4a	243 (47.6)	405 (69.1)		224 (62.9)	224 (65.3)	
pN stage			0.036			0.163
N0	155 (30.4)	162 (27.6)		67 (19.4)	92 (26.6)	
N1	78 (15.3)	65 (11.1)		61 (17.6)	48 (13.9)	
N2	91 (17.8)	95 (16.2)		61 (17.6)	56 (16.2)	
N3a	109 (21.4)	164 (28.0)		88 (25.4)	91 (26.3)	
N3b	77 (15.1)	100 (17.1)		69 (19.9)	59 (17.1)	
pTNM stage			< 0.001			0.735
IA	60 (11.8)	49 (8.4)		29 (8.4)	30 (8.7)	
IB	34 (6.7)	40 (6.8)		17 (4.9)	21 (6.1)	
IIA	49 (9.6)	28 (4.8)		13 (3.8)	22 (6.4)	
IIB	68 (13.3)	74 (12.6)		40 (11.6)	40 (11.6)	
IIIA	70 (13.7)	56 (9.6)		49 (14.2)	41 (11.8)	
IIIB	97 (19.0)	109 (18.6)		69 (19.9)	68 (19.7)	
IIIC	132 (25.9)	230 (39.2)		129 (37.3)	124 (35.8)	
Postoperative chemotherapy			0.170			0.287
Yes	413 (81.0)	493 (84.1)		310 (89.6)	301 (87.0)	
No	97 (19.0)	93 (15.9)		36 (10.4)	45 (13.0)	
No. of comorbidities			0.794			0.317
0	362 (71.0)	418 (71.3)		241 (69.7)	249 (72.0)	
1	109 (21.3)	117 (20.0)		82 (23.7)	68 (19.7)	
2	35 (6.9)	43 (7.3)		21 (6.0)	23 (6.6)	
≥ 3	4 (0.8)	8 (1.4)		2 (0.6)	6 (1.7)	

BMI: body mass index; No. of comorbidities: number of comorbidities

The propensity scores were calculated using a logistic regression model to balance the following covariates: age, gender, body mass index (BMI), comorbidities, tumor location, histologic type, pT stage and pN stage. Finally, 692 patients (346 patients who underwent LATG and 346 patients who underwent OTG) were selected for analysis. After propensity score matching, age, gender, BMI, tumor location, tumor size, histologic type, pT stage, pN stage, pTNM stage and the number of comorbidities did not differ significantly between the two groups.

### Surgical results

Before propensity score matching, the LATG group exhibited a significantly shorter operation time, less blood loss, shorter time to first flatus, shorter time to starting a liquid diet and a higher number of retrieved lymph nodes than the OTG group (*P* < 0.05).

After propensity score matching, the LATG group had a significantly shorter mean operation time (205.8 ± 63.0 min vs. 282.0 ± 64.0 min, *P* < 0.001), less mean blood loss (90 ml vs. 209 ml, *P* < 0.001), a shorter mean time to first flatus (3.7 vs. 4.0, *P* < 0.001) and a higher number of retrieved lymph nodes (31.6 vs. 29.7, *P* = 0.017) than the OTG group (Table 2).

**Table 2:** short-term outcomes of LATG and OTG groups

Variable	All patients (*n* = 1096)			Patients propensity matching (*n* = 692)		
	LATG(*n* = 510)	OTG(*n* = 586)	*P*	LATG(*n* = 346)	OTG(*n* = 346)	*P*
Operation time (min)	201.3 ± 62.6	277.6 ± 63.5	< 0.001	205.8 ± 63.0	282.0 ± 64.0	< 0.001
Estimated blood loss (ml)	85.8 ± 117.9	208.1 ± 164.4	< 0.001	90.0 ± 130.0	209.0 ± 172.4	< 0.001
No. of retrieved lymph nodes	31.6 ± 11.1	29.9 ± 10.8	0.013	31.6 ± 10.3	29.7 ± 10.8	0.017
Time to first flatus (days)	3.7 ± 1.0	4.0 ± 1.0	< 0.001	3.7 ± 1.0	4.0 ± 1.0	< 0.001
Time to start liquid diet(days)	4.4 ± 1.2	4.6 ± 1.0	0.045	4.4 ± 1.2	4.6 ± 1.10	0.067
Time to start soft diet (days)	7.6 ± 0.9	7.6 ± 0.7	0.988	7.6 ± 0.9	7.6 ± 0.7	0.850
Postoperative hospital stay (days)	15.2 ± 10.7	15.9 ± 7.9	0.203	15.7 ± 12.4	15.9 ± 8.1	0.859

No. of retrieved lymph nodes: number of retrieved lymph nodes

### Postoperative complications of the LATG group and OTG group

The postoperative complication rates of the LATG group and OTG group were 14.9% and 21.0% (*P* = 0.009), respectively. After propensity matching, the postoperative complication rates of the LATG group and OTG group were 15.0% and 22.8% (*P* = 0.009), respectively. The rates of category I–II complications and the rates of category III-IV complications did not significantly differ (*P* > 0.05).

None of the patients in the LATG group died, whereas mortality was 1.2% in the OTG group before propensity matching (*P* = 0.017). No significant intergroup differences were found in morbidity after propensity matching (Table 3).

**Table 3:** Postoperative complications of LATG and OTG groups

Variable	All patients(*n* = 1096)			Patients propensity matching (*n* = 692)		
	LATG(*n* = 510)	OTG(*n* = 586)	*P*	LATG(*n* = 346)	OTG(*n* = 346)	*P*
**Total complication**	76(14.9%)	123(21.0%)	0.009	52(15.0%)	79(22.8)	0.009
**I–II complication**	51(10.0%)	76(13.0%)	0.126	37(10.7%)	51(14.7%)	0.110
Pneumonia	17	24		14	19	
Anastomotic leakage	5	6		3	4	
Abdominal infection	8	12		5	8	
Ileus	5	4		4	3	
Intra-abdominal bleeding	0	1		0	1	
Lymphatic leakage	3	4		2	2	
Pancreatic fistula	1	1		0	0	
Wound infection	4	11		3	7	
Others	8	13		6	7	
**III–IV complication**	15(2.9%)	15(2.6%)	0.699	10(2.9%)	6(1.7%)	0.312
Pneumonia	7	10		3	7	
Anastomotic leakage	4	5		3	4	
Abdominal infection	2	8		1	6	
Ileus	1	3		1	2	
Intra-abdominal bleeding	4	4		3	2	
Wound infection	2	4		1	2	
Lymphatic leakage	1	2		1	1	
Others	4	4		2	2	
**Death**	0	7(1.2%)	0.017	0	2(0.6%)	0.499

### Risk factors of postoperative morbidity

A univariate analysis showed that age (*P* = 0.030), operative approach (*P* = 0.009) and comorbidities (*P* = 0.023) were closely related to postoperative complications. According to the multivariate analysis, the operative approach (*P* = 0.009) and comorbidities (*P* = 0.035) were independent risk factors for postoperative morbidity (Table 4).

**Table 4:** Risk factors of postoperative morbidity

Variable	Postoperative morbidity		Univariate analysis *P*	Multivariate analysis		
	No(*n* = 561)	Yes(*n* = 131)		OR	95%CI	*P*
Age	60.7 ± 10.1	62.8 ± 9.9	0.030	1.016		0.135
Gender			0.135			
Male	438(78.1)	70(84.0)				
Female	123(21.9)	18(16.0)				
BMI(kg/m2)	22.0 ± 2.6	21.8 ± 2.5	0.201			
Tumor Location			0.422			
Upper	302(53.8)	77(58.8)				
Middle	227(40.5)	45(34.4)				
Total	32(5.7)	9(6.9)				
Operative approach			0.009	0.596		0.009
LATG	294(52.4)	52(39.7)				
OTG	267(47.6)	79(60.3)				
Tumor size(cm)	6.1 ± 2.9	5.7 ± 2.6	0.191			
pT stage			0.270			
T1	51(9.1)	17(13.0)				
T2	44(7.8)	6(4.6)				
T3	99(17.7)	27(20.6)				
T4a	367(65.4)	81(61.8)				
pN stage			0.423			
N0	127 (22.6)	32(24.4)				
N1	83(14.8)	26(19.8)				
N2	95(16.9)	22(16.8)				
N3a	146(26.0)	33(25.2)				
N3b	110(19.6)	18(13.7)				
pTNM stage			0.402			
IA	48(8.6)	11(8.4)				
IB	27(4.8)	11(6.4)				
IIA	30(5.3)	5(3.8)				
IIB	62(11.1)	18(13.7)				
IIIA	72(12.8)	18(13.7)				
IIIB	108(19.3)	29(22.1)				
IIIC	214(38.1)	39(29.8)				
No. of comorbidities			0.023	1.527		0.035
0	418(73.7)	82(62.6)				
1–2	138(25.6)	47(35.9)				
≥ 3	5(0.9)	2(1.5)				
No. of retrieved lymph nodes	30.9 ± 10.7	29.6 ± 10.2	0.212			
Operative time (min)	241.8 ± 71.5	252.9 ± 83.3	0.121			
Estimated blood loss (ml)	144.5 ± 155.8	171.1v193.0	0.094			

OR: odds ratio; CI: confidence interval

### Survival after surgery

**Figure 1:**
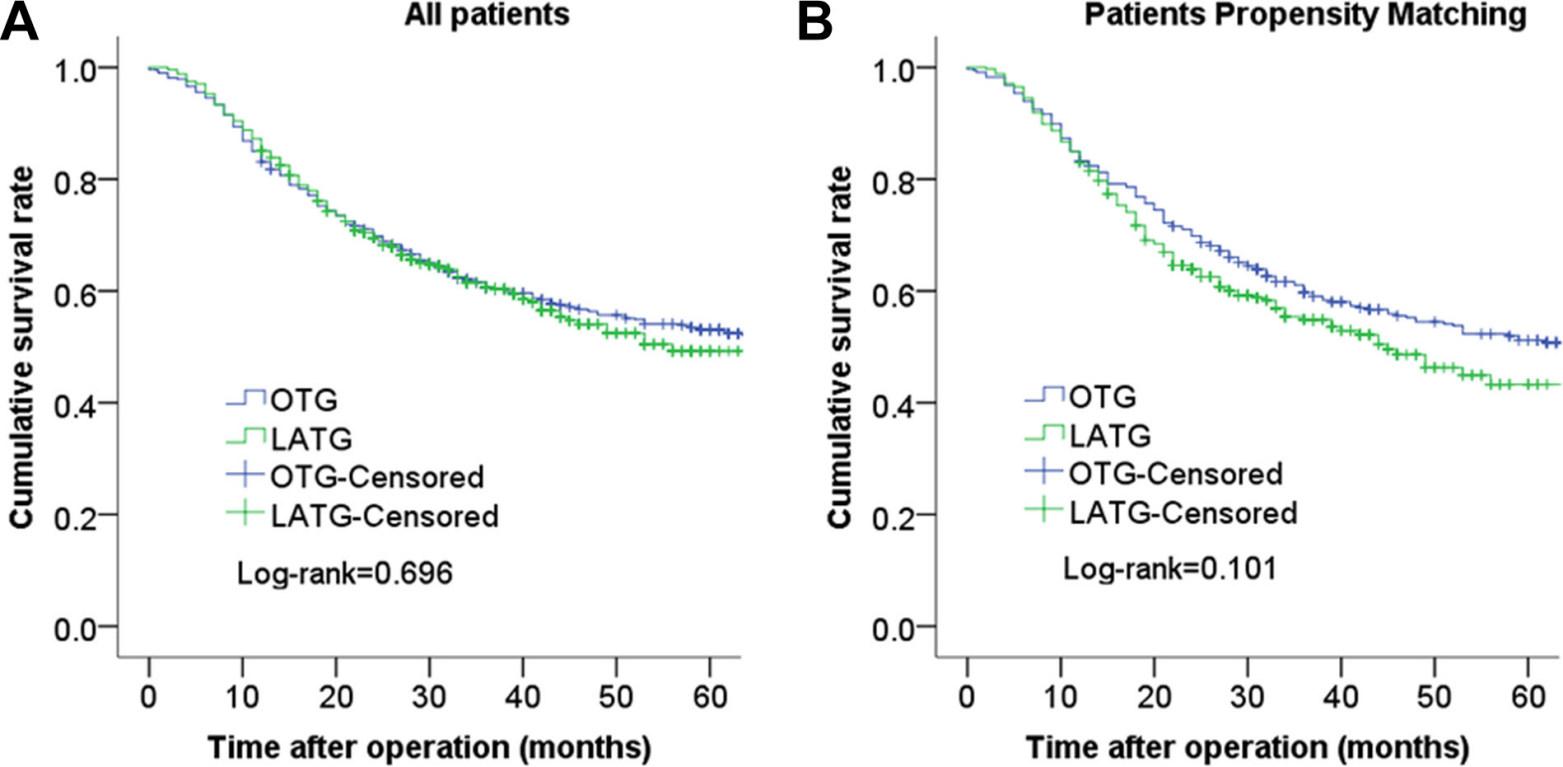
Comparison of cumulative survival rates between LATG and OTG before propensity matching (*P* = 0.696) (A) and cumulative survival rates between LATG and OTG after propensity matching (*P* = 0.101) (B) There was no statistically significant difference between the two groups.

**Figure 2:**
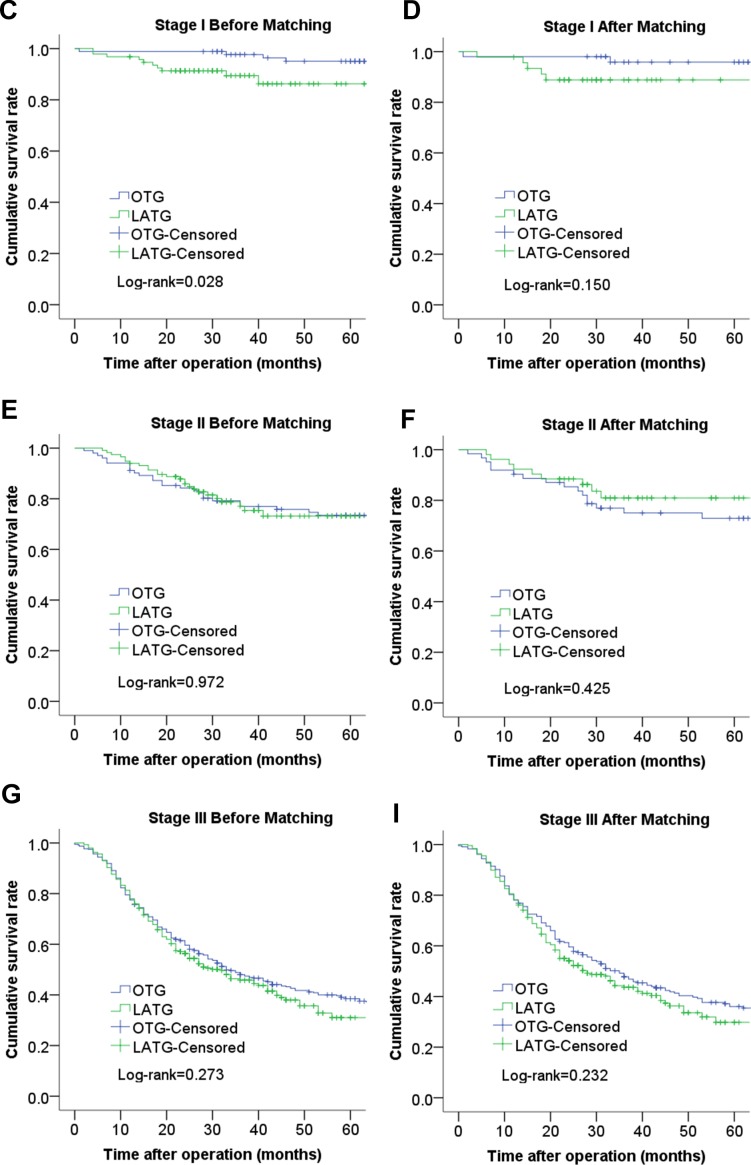
Comparison of cumulative survival rates between LATG and OTG according to cancer stage (**C**). stage I before matching; (**D**) stage I after matching; (**E**) stage II before matching; (**F**) stage II after matching; (**G**) stage III before matching; (**H**) stage III after matching;

The median follow-up period was 45 months (range 1–115 months). The 3-year cumulative survival rates of the LATG group and OTG group were 61.5% and 60.7% (*P* = 0.696) before propensity matching and 59.7% and 54.9% (*P* = 0.101) after propensity matching, respectively (Figure 1). The 3-year cumulative survival rates of the LATG group and OTG group were 89.9% and 97.7% for patients with stage I disease (*P* = 0.028), and the 3-year cumulative survival rates did not significantly differ between patients with stage II and stage III disease (*P* > 0.05). After propensity score matching, the analysis of the cumulative survival curve did not show significant difference for any cancer stage (Figure 2).

## DISCUSSION

Laparoscopic gastrectomy is an acceptable alternative to conventional open gastrectomy for patients with ECG not only because the two procedures result in similar survival and recurrence [8, 9] but also because patients tend to experience better early postoperative outcomes than those undergoing open gastrectomy [2–4]. In recent years, several experienced institutions have reported favorable long-term oncological outcomes as well as the improved technical feasibility and safety of LADG for the treatment of advanced gastric cancer compared with conventional open gastrectomy [6, 10, 11]. However, few studies have examined LATG due to extended lymph node detection and complex digestive construction. Moreover, these retrospective studies have primarily examined EGC in small samples [12–17], and randomized controlled clinical trials have not been reported. Therefore, our institution enrolled a large number of patients to evaluate the feasibility, safety and clinical efficacy of LATG before conducting randomized controlled clinical trials that evaluate the efficacy of LATG for the treatment of gastric cancer. Specifically, the propensity score-matching method was used to reduce bias.

In previous studies of LDG and ODG, the main advantages of laparoscopic gastrectomy over conventional open surgery included less blood loss, a smaller skin incision, a shorter time to ambulation, a shorter time to a liquid diet and a shorter postoperative hospital stay [5, 18] as well as the ability to visualize finer structures due to laparoscopic amplification. However, LG is also associated with disadvantages, such as increased cost, the need for a surgeon skilled in laparoscopic techniques and a longer learning curve [19]. However, few studies have examined the minimal invasiveness of laparoscopic total gastrectomy. The results of our study provide new evidence of the minimally invasive nature of laparoscopic total gastrectomy. Specifically, the LATG group in our study exhibited a significantly reduced mean estimated blood loss and shorter times to flatus and liquid diet consumption compared to the OTG group. Some studies [12–14, 20, 21] reported that the operation time was longer for laparoscopic gastrectomy than conventional open gastrectomy. Nevertheless, improvements in laparoscopic techniques, surgical instruments and the accumulation of experience have reduced the operation time for laparoscopic gastrectomy [22]. Specifically, Kunisaki et al. [23] found that the accumulation of experience reduced the operation time; the operation time associated with LAG was significantly shorter in a medical group performing 81 to 100 surgeries than in a group performing 1 to 20 surgeries (221.6 min vs. 351.2 min, P>0.05). Thus, laparoscopic gastrectomy did not require more time than conventional open gastrectomy when performed by an experienced surgeon [13], and the incision required for laparoscopic gastrectomy is preferred to that required for open gastrectomy. Additionally, our institution uses a systematic laparoscopic gastrectomy procedure [24] that simplifies this complex procedure, improves the efficiency of laparoscopic surgery and shortens the operation time of LATG compared to conventional OTG. Our data showed that more lymph nodes were dissected in the LATG group more than in the OTG group, and the average number of lymph nodes harvested exceeded 30 in both groups. The key to lymph node detection is being skilled in laparoscopic techniques and a good anatomical sense under the laparoscope. A laparoscope can help the surgeon dissect lymph nodes under special anatomical structures because the laparoscope can amplify the vasculature, nerves, fascia and other structures. In addition, our center took procedural operative steps [24–26] to dissect the perigastric lymph nodes. Thus, our laparoscopic surgical procedure is preferred over open gastrectomy for AGC lymphadenectomy. This result suggested that the number of retrieved lymph nodes was comparable for LATG and OTG.

Postoperative morbidity and mortality are important objective factors to evaluate technical feasibility. Specifically, the postoperative complications rate of LATG has been reported to range from 8.5% to 32% [12, 14, 16, 20, 21], and these differences may be due to differences in the experience of surgeons as well as differences in the definitions and classifications of postoperative complications. In recent years, many scholars have recommended the Clavien-Dindo classification system has been recommended to define and grade postoperative complications. This system was tested in a large cohort of patients who underwent general surgery, and the acceptability of this classification system was assessed with an international survey, which demonstrated that this system appears to be a reliable tool for quality assessment in surgery [27, 28]. In our study, the postoperative complication rates of the LATG group and OTG group were 14.9% and 21.0% (*P* = 0.009), respectively, and the mortality rates according to the Clavien-Dindo system did not significantly differ between groups after propensity matching. In our multivariate analysis, operative approach and comorbidities were identified as independent factors related to postoperative complications. Moreover, our study confirmed that comorbidities may increase the postoperative complications rate, which has been reported in a previous study [29]. These results suggested that the safety and feasibility of LATG and OTG were comparable.

Long-term survival rates are important elements for assessing oncological safety. Specifically, laparoscopic gastrectomy can only be accepted as an alternative approach to open gastrectomy if similar long-term outcomes can be achieved. Previous studies showed that the long-term survival rates of patients who underwent LATG were similar to those of patients who underwent OTG, but these reports primarily examined small cohorts of patients who underwent LATG for EGC. A case control study [14] reported that the long-term outcomes of LATG were similar to those of open OTG for patients with AGC. Moreover, Kim et al. [19] found that the long-term oncologic outcomes of laparoscopic gastrectomy for 238 patients with AGC were consistent with those of open gastrectomy in a case-controlled and case-matched study. Although the oncological results of the LATG group were similar to those of the OTG group for the pre-matched patients, the cumulative survival rate of patients with stage I gastric cancer in the LATG group was significantly lower than that in the OTG group. This difference may be due to the heterogeneity of the two groups. Specifically, the proportion of undifferentiated histology was higher in the LATG group than the OTG group, and the cumulative survival rate did not significantly differ between the two groups after the propensity score-matched analysis. These results suggested that the long-term outcomes achieved by LATG and OTG were comparable.

Our study was also subject to several limitations. We conducted the study at a single center in a retrospective manner, and patients who experienced recurrence were not described in detail due to incomplete follow-up results. Nonetheless, to the best of our knowledge, this study examined the largest number of patients with advanced gastric cancer who have undergone LATG at a high-volume center and used the propensity score-matching method to reduce bias.

In conclusion, LATG is a safe and reliable procedure for the treatment of gastric cancer when conducted by experienced surgeons at high-volume institutions. The morbidity and mortality of LATG is acceptable when compared with OTG, and oncologic outcomes are similar in both groups. Additional randomized controlled clinical trials can be conducted to provide valuable evidence of the safety and efficacy of LATG in treating gastric cancer.

## MATERIALS AND METHODS

### Patients and methods

Between January 2005 and June 2012, 1096 patients who were diagnosed with primary gastric cancer and underwent total gastrectomy for their gastric cancer were identified using a prospectively maintained gastric cancer database at the Department of Gastric Surgery, Fujian Medical University Union Hospital, China. OTG and LATG were concurrently performed. Ultimately, 510 patients were treated with LATG, and 586 patients were treated with OTG. The inclusion criteria were defined as follows: histologically proven gastric carcinoma, no distant metastasis, tumors located in the upper two-thirds of the stomach or throughout the stomach, gastric cancer patients who were treated with a total gastrectomy and an R0 resection performed according to the surgical and pathological findings. The exclusion criteria were defined as follows: stage T4b or distant metastasis, treatment with preoperative chemotherapy or radiotherapy, a lack of a pathological diagnosis, remnant gastric cancer, palliative gastrectomy or an emergency operation with bleeding or perforation. Lymph node dissection was performed according to the Japanese gastric cancer treatment guidelines [30, 31]. The data on the extent of lymphadenectomy were retrospectively revised according to a recently published version of these guidelines [31]. Tumor staging was based on the 7th edition of the pathological (pTNM) classification of the Union for International Cancer Control (UICC)/American Joint Committee on Cancer (AJCC). The comorbidities were staged according to the Charlson comorbidity index [32]. The severity of postoperative complications was classified according to the Clavien-Dindo classification system [27]. All patients were informed of the possible complications and given a full detailed explanation of each surgical method as well as the advantages and disadvantages of LATG versus conventional OTG. All patients selected their surgery, and written informed consent was obtained prior to the surgery. Adjuvant chemotherapy using 5-fluorouracil (5-FU)-based regimens (mostly Oxaliplatin with either Xeloda or S1) was recommended to the majority of patients with advanced gastric cancer.

### Follow-up

Specially trained researchers used outpatient records, visitation, letters and telephone calls to follow up with patients after their operation. Patients were followed up every 3 months for 2 years and then every 6 months from postoperative years 3 to 5. All patients were followed for at least 3 years after surgery. Survival time was defined as the time from surgery to either death or the final follow-up date of March 2016.

### Statistical analysis

Statistical analysis was performed using SPSS v13.0 for Windows (SPSS Inc., Chicago, IL). Categorical variables were analyzed using the Chi-squared or Fisher’s exact test, whereas continuous variables were analyzed using either the unpaired Student’s *t*-test or the Mann-Whitney U test. Multiple factor logistic regression models [33] were used to calculate the propensity score for each patient, and we imposed a caliper width of 0.02 of the standard deviation of the logistic of the propensity score. Patients in the LATG group were individually matched to patients in the OTG group according to the nearest neighbor matching principle and the non-replacement principle (i.e., a single case cannot be used multiple times). Multivariate analyses were performed by binary logistic multiple regression tests to identify independent risk factors for postoperative morbidity. Cumulative survival rates were estimated using the Kaplan-Meier method and compared with the log-rank test. Two-sided *P* values less than 0.05 were considered to be significant.

## References

[1] Kitano S, Iso Y, Moriyama M, Sugimachi K (1994). Laparoscopy-assisted Billroth I gastrectomy. Surg Laparosc Endosc.

[2] Sakuramoto S, Yamashita K, Kikuchi S, Futawatari N, Katada N, Watanabe M, Okutomi T, Wang G, Bax L (2013). Laparoscopy versus open distal gastrectomy by expert surgeons for early gastric cancer in Japanese patients: short-term clinical outcomes of a randomized clinical trial. Surg Endosc.

[3] Zeng YK, Yang ZL, Peng JS, Lin HS, Cai L (2012). Laparoscopy-assisted versus open distal gastrectomy for early gastric cancer: evidence from randomized and nonrandomized clinical trials. Ann Surg.

[4] Deng Y, Zhang Y, Guo TK (2015). Laparoscopy-assisted versus open distal gastrectomy for early gastric cancer: A meta-analysis based on seven randomized controlled trials. Surg Oncol.

[5] Hu Y, Huang C, Sun Y, Su X, Cao H, Hu J, Xue Y, Suo J, Tao K, He X, Wei H, Ying M, Hu W (2016). Morbidity and Mortality of Laparoscopic Versus Open D2 Distal Gastrectomy for Advanced Gastric Cancer: A Randomized Controlled Trial. J Clin Oncol.

[6] Park YK, Yoon HM, Kim YW, Park JY, Ryu KW, Lee YJ, Jeong O, Yoon KY, Lee JH, Lee SE, Yu W, Jeong SH, Kim T (2017 Mar 15). Laparoscopy-Assisted versus Open D2 Distal Gastrectomy for Advanced Gastric Cancer: Results from a Randomized Phase II Multicenter Clinical Trial (COACT 1001). Ann Surg.

[7] Katai H, Sasako M, Fukuda H, Nakamura K, Hiki N, Saka M, Yamaue H, Yoshikawa T, Kojima K, Group JGCSS (2010). Safety and feasibility of laparoscopy-assisted distal gastrectomy with suprapancreatic nodal dissection for clinical stage I gastric cancer: a multicenter phase II trial (JCOG 0703). Gastric Cancer.

[8] Lee JH, Nam BH, Ryu KW, Ryu SY, Park YK, Kim S, Kim YW (2015). Comparison of outcomes after laparoscopy-assisted and open total gastrectomy for early gastric cancer. Br J Surg.

[9] Honda M, Hiki N, Kinoshita T, Yabusaki H, Abe T, Nunobe S, Terada M, Matsuki A, Sunagawa H, Aizawa M, Healy MA, Iwasaki M, Furukawa TA (2016). Long-term Outcomes of Laparoscopic Versus Open Surgery for Clinical Stage I Gastric Cancer: The LOC-1 Study. Ann Surg.

[10] Gordon AC, Kojima K, Inokuchi M, Kato K, Sugihara K (2013). Long-term comparison of laparoscopy-assisted distal gastrectomy and open distal gastrectomy in advanced gastric cancer. Surg Endosc.

[11] Shuang J, Qi S, Zheng J, Zhao Q, Li J, Kang Z, Hua J, Du J (2011). A case-control study of laparoscopy-assisted and open distal gastrectomy for advanced gastric cancer. J Gastrointest Surg.

[12] Kim KH, Kim YM, Kim MC, Jung GJ (2013). Is laparoscopy-assisted total gastrectomy feasible for the treatment of gastric cancer? A case-matched study. Dig Surg.

[13] Kim HS, Kim BS, Lee IS, Lee S, Yook JH, Kim BS (2013). Comparison of totally laparoscopic total gastrectomy and open total gastrectomy for gastric cancer. J Laparoendosc Adv Surg Tech A.

[14] Bo T, Peiwu Y, Feng Q, Yongliang Z, Yan S, Yingxue H, Huaxing L (2013). Laparoscopy-assisted vs. open total gastrectomy for advanced gastric cancer: long-term outcomes and technical aspects of a case-control study. J Gastrointest Surg.

[15] Shim JH, Oh SI, Yoo HM, Jeon HM, Park CH, Song KY (2013). Short-term outcomes of laparoscopic versus open total gastrectomy: a matched-cohort study. Am J Surg.

[16] Lee SR, Kim HO, Son BH, Shin JH, Yoo CH (2014). Laparoscopic-assisted total gastrectomy versus open total gastrectomy for upper and middle gastric cancer in short-term and long-term outcomes. Surg Laparosc Endosc Percutan Tech.

[17] Tuttle R, Hochwald SN, Kukar M, Ben-David K (2016). Total laparoscopic resection for advanced gastric cancer is safe and feasible in the Western population. Surg Endosc.

[18] Katai H, Mizusawa J, Katayama H, Takagi M, Yoshikawa T, Fukagawa T, Terashima M, Misawa K, Teshima S, Koeda K, Nunobe S, Fukushima N, Yasuda T (2016 Oct 7). Short-term surgical outcomes from a phase III study of laparoscopy-assisted versus open distal gastrectomy with nodal dissection for clinical stage IA/IB gastric cancer: Japan Clinical Oncology Group Study JCOG0912. Gastric Cancer.

[19] Kim HH, Han SU, Kim MC, Hyung WJ, Kim W, Lee HJ, Ryu SW, Cho GS, Song KY, Ryu SY (2014). Long-term results of laparoscopic gastrectomy for gastric cancer: a large-scale case-control and case-matched Korean multicenter study. J Clin Oncol.

[20] Eom BW, Kim YW, Lee SE, Ryu KW, Lee JH, Yoon HM, Cho SJ, Kook MC, Kim SJ (2012). Survival and surgical outcomes after laparoscopy-assisted total gastrectomy for gastric cancer: case-control study. Surg Endosc.

[21] Lee MS, Lee JH, Park DJ, Lee HJ, Kim HH, Yang HK (2013). Comparison of short- and long-term outcomes of laparoscopic-assisted total gastrectomy and open total gastrectomy in gastric cancer patients. Surg Endosc.

[22] Kim MC, Jung GJ, Kim HH (2005). Learning curve of laparoscopy-assisted distal gastrectomy with systemic lymphadenectomy for early gastric cancer. World J Gastroenterol.

[23] Kunisaki C, Makino H, Yamamoto N, Sato T, Oshima T, Nagano Y, Fujii S, Akiyama H, Otsuka Y, Ono HA, Kosaka T, Takagawa R, Shimada H (2008). Learning curve for laparoscopy-assisted distal gastrectomy with regional lymph node dissection for early gastric cancer. Surg Laparosc Endosc Percutan Tech.

[24] Huang CM, Chen QY, Lin JX, Zheng CH, Li P, Xie JW (2014). Huang’s three-step maneuver for laparoscopic spleen-preserving No. 10 lymph node dissection for advanced proximal gastric cancer. Chin J Cancer Res.

[25] Huang CM, Chen QY, Lin JX, Zheng CH, Li P, Xie JW, Wang JB, Lu J, Yang XT (2015). Laparoscopic Suprapancreatic Lymph Node Dissection for Advanced Gastric Cancer Using a Left-Sided Approach. Ann Surg Oncol.

[26] Huang CM, Chen QY, Lin JX, Zheng CH, Li P, Xie JW, Wang JB, Lu J, Yang XT (2014). Laparoscopic spleen-preserving no. 10 lymph node dissection for advanced proximal gastric cancer using a left approach. Ann Surg Oncol.

[27] Dindo D, Demartines N, Clavien PA (2004). Classification of surgical complications: a new proposal with evaluation in a cohort of 6336 patients and results of a survey. Ann Surg.

[28] Clavien PA, Sanabria JR, Strasberg SM (1992). Proposed classification of complications of surgery with examples of utility in cholecystectomy. Surgery.

[29] Yu J, Hu J, Huang C, Ying M, Peng X, Wei H, Jiang Z, Du X, Liu Z, Liu H, Li G, Chinese Laparoscopic Gastrointestinal Surgery Study G (2013). The impact of age and comorbidity on postoperative complications in patients with advanced gastric cancer after laparoscopic D2 gastrectomy: results from the Chinese laparoscropic gastrointestinal surgery study (CLASS) group. Eur J Surg Oncol.

[30] Nakajima T (2002). Gastric cancer treatment guidelines in Japan. Gastric Cancer.

[31] Japanese Gastric Cancer A. Japanese gastric cancer treatment guidelines 2010 (ver. 3) (2011). Gastric Cancer.

[32] Charlson M, Wells MT, Ullman R, King F, Shmukler C (2014). The Charlson comorbidity index can be used prospectively to identify patients who will incur high future costs. PLoS One.

[33] D’Agostino RB (1998). Propensity score methods for bias reduction in the comparison of a treatment to a non-randomized control group. Stat Med.

